# Boosting Photoelectrochemical Water Splitting via InPO_x_-Coated TiO_2_ Nanowire Photoanodes

**DOI:** 10.3390/molecules30173482

**Published:** 2025-08-25

**Authors:** Ying-Chu Chen, Heng-Yi Lin, Yu-Kuei Hsu

**Affiliations:** 1 Department of Chemical Engineering & Biotechnology, National Taipei University of Technology, Taipei City 10608, Taiwan; 2 Department of Opto-Electronic Engineering, National Dong Hwa University, No. 1, Sec. 2, Da Hsueh Road, Shoufeng, Hualien 97401, Taiwan

**Keywords:** TiO_2_, InPO_x_, Core–Shell Heterojunction, Photoelectrochemical Water Splitting, Bandgap Engineering

## Abstract

A hierarchical photoanode composed of amorphous indium phosphate (InPO_x_)-coated titanium dioxide nanowires (TiO_2_ NWs) was successfully fabricated via a hydrothermal method followed by dip-coating and thermal phosphidation. Structural characterization revealed the formation of a uniform InPO_x_ shell on the surface of vertically aligned TiO_2_ NWs, without altering their 1D morphology. X-ray photoelectron spectroscopy confirmed the incorporation of phosphate species and the presence of oxygen vacancies, which contribute to enhanced interfacial charge dynamics. Photoelectrochemical (PEC) measurements demonstrated that the InPO_x_/TiO_2_ NWs significantly improved photocurrent density, with the 0.1 M InCl_3_-derived sample achieving 0.36 mA·cm^−2^ at 1.0 V—an enhancement of approximately 928% over pristine TiO_2_. This enhancement is attributed to improved charge separation and injection efficiency (91%), as well as reduced interfacial resistance verified by electrochemical impedance spectroscopy. Moreover, the Mott–Schottky analysis indicated a four-order increase in carrier density due to the InPO_x_ shell. The modified electrode also exhibited superior stability under continuous illumination for 3 h. These findings highlight the potential of amorphous InPO_x_ as an effective cocatalyst for constructing efficient and durable TiO_2_-based photoanodes for solar-driven water-splitting applications.

## 1. Introduction

With the growing global demand for clean energy and sustainable hydrogen production, significant efforts have been devoted to utilizing solar energy for water splitting. However, the intermittent and decentralized nature of solar irradiation poses practical challenges to energy conversion. Among various strategies, photoelectrochemical (PEC) water splitting has emerged as a promising approach for direct solar-to-hydrogen conversion [1]. To date, various n-type semiconductors—such as TiO_2_, Fe_2_O_3_, BiVO_4_, and ZnO—have been investigated as photoanode materials for PEC water oxidation [2,3,4,5]. TiO_2_, in particular, has attracted widespread attention due to its chemical stability, abundance, and appropriate band-edge positions. Nevertheless, its wide bandgap (~3.2 eV for anatase), short charge diffusion length, and sluggish surface reaction kinetics significantly limit its PEC efficiency. One viable strategy to overcome these drawbacks is the construction of composite photoelectrodes with optimized compositions and nanostructures. For example, forming semiconductor heterojunctions or decorating the surface with oxygen evolution reaction (OER) cocatalysts can effectively enhance charge separation and lower the reaction barriers [6]. While incorporating narrow-bandgap semiconductors or cocatalysts onto TiO_2_ surfaces is a common tactic, such modifications may compromise its intrinsic UV light absorption [7,8,9]. Alternatively, coupling TiO_2_ with a wide bandgap cocatalyst offers a promising path to enhance charge separation without sacrificing light harvesting. Recently, Gao et al. reported improved PEC performance by combining In_2_S_3_ nanosheets with InPO_x_ overlays. InPO_x_ compounds, such as InPO_4_, exhibit wide bandgaps (e.g., ~4.5 eV), making them suitable candidates for this approach [10,11]. In this study, we deposited InPO_x_ shells onto TiO_2_ nanowires via dip-coating followed by thermal phosphidation. Compared to bare TiO_2_, the resulting InPO_x_/TiO_2_ photoanode exhibited a tenfold increase in photocurrent at 1 V vs. Ag/AgCl in 0.5 M Na_2_SO_4_, indicating substantially improved PEC performance. This work not only advances the design of efficient photoelectrodes through band structure modulation but also opens new avenues for 3D hierarchical nanomaterials in solar energy and electronic applications.

## 2. Results and Discussion

### 2.1. Structure and Composition Characterization

**Figure 1:**
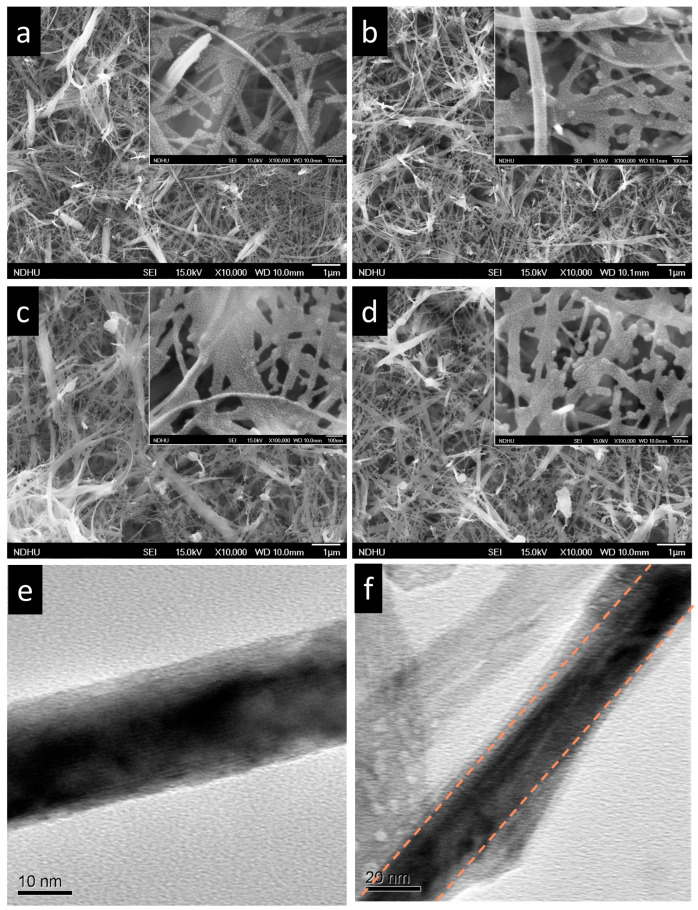
FESEM images of (**a**) pristine TiO_2_ NWs and InPO_x_/TiO_2_ NWs with InCl_3_ concentrations of (**b**) 0.05 M, (**c**) 0.1 M, and (**d**) 0.15 M. High-magnification SEM insets. TEM images of (**e**) pristine TiO_2_ NWs and (**f**) InPO_x_/TiO_2_ NWs with 0.1 M InCl_3_.

The morphological evolution of the InPO_x_/TiO_2_ nanowires (NWs) was investigated using scanning electron microscopy (SEM). As shown in Figure 1a, the pristine TiO_2_ NWs synthesized via the hydrothermal method exhibit a dense, wire-like structure that uniformly covers a large area of the Ti foil substrate. The NWs have diameters ranging from 20 to 150 nm and lengths extending to several tens of micrometers. Figure 1b–d present the surface morphologies of the TiO_2_ NWs after deposition of the InPO_x_ layer using InCl_3_ solutions with concentrations of 0.05, 0.1, and 0.15 M, respectively. The 1D morphology and density of the NWs remain unchanged across all samples. The mean nanowire diameter increases from 26 nm (pristine) to 33 nm (0.05 M), 42 nm (0.1 M), and 47 nm (0.15 M). However, a noticeable increase in both nanowire diameter and surface roughness is observed, indicating the successful formation of an InPO_x_ shell layer on the TiO_2_ NWs.

To further confirm the core–shell structure, transmission electron microscopy (TEM) images of the pristine TiO_2_ NWs and InPO_x_/TiO_2_ NWs (prepared with 0.1 M InCl_3_) are shown in Figure 1e and Figure 1f, respectively. The pristine TiO_2_ NWs exhibit smooth surfaces, whereas the InPO_x_/TiO_2_ NWs display a rougher surface with an additional layer. This overlayer corresponds to an InPO_x_ shell with an estimated thickness of 2–15 nm, validating the successful deposition of the amorphous coating.

**Figure 2:**
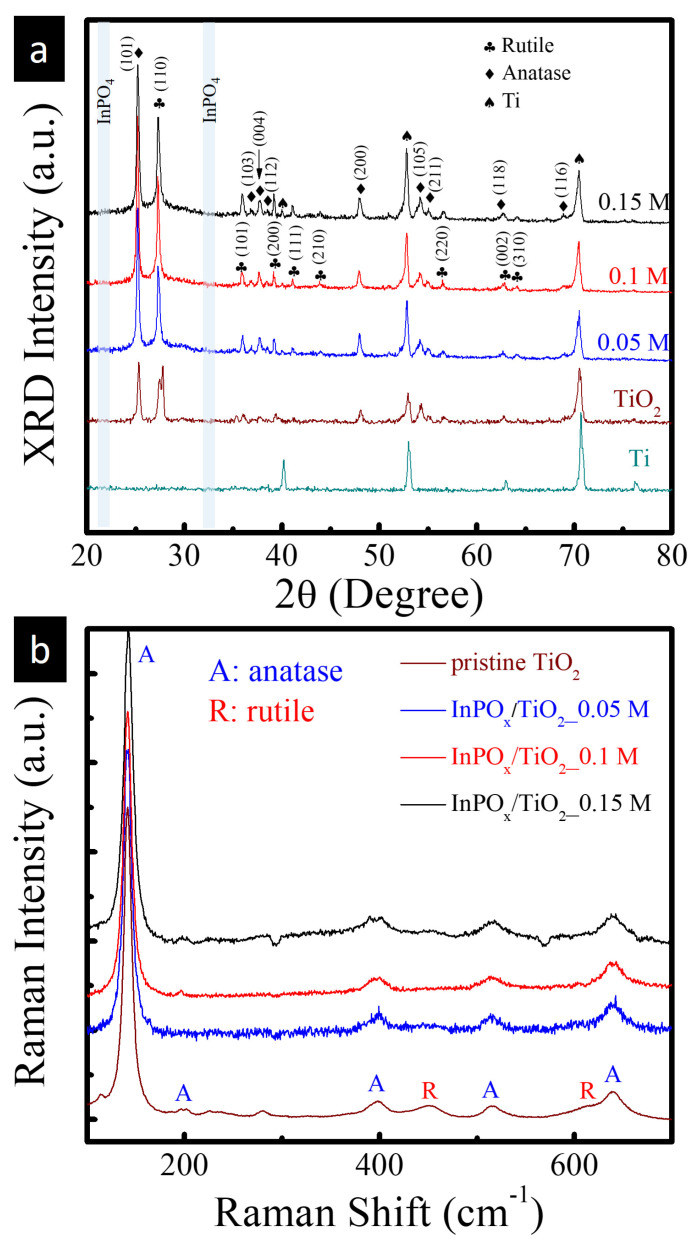
(**a**) XRD patterns and (**b**) Raman spectra of InPO_x_/TiO_2_ NW samples with InCl_3_ concentrations of 0.05 M, 0.1 M and 0.15 M.

X-ray diffraction (XRD) was employed to investigate the crystallographic structure of the InPO_x_/TiO_2_ heterostructure, as shown in Figure 2a. For comparison, XRD patterns of the bare TiO_2_ NWs and the Ti foil substrate are also included. Both the pristine and InPO_x_-modified TiO_2_ NW samples exhibit diffraction peaks that can be indexed to two TiO_2_ polymorphs. The peaks labeled with diamonds correspond to anatase TiO_2_ (JCPDS No. 21-1272), including the (101), (004), and (200) reflections. The peaks marked with plum blossom symbols are attributed to the rutile TiO_2_ phase (JCPDS No. 88-1175), corresponding to the (110), (101), and (002) planes. Lattice parameters of anatase (a = 3.785 Å, c = 9.514 Å) and rutile (a = 4.593 Å, c = 2.958 Å) were extracted. These values are in excellent agreement (within ±0.3%) with the standard phases, confirming phase purity and structural stability during InPO_x_ deposition. The average crystallite sizes of the (101) anatase and (110) rutile reflections are ~22 nm and ~28 nm, respectively. Aside from these, diffraction peaks arising from the underlying Ti substrate (marked with spades) are also observed. Importantly, no additional diffraction peaks corresponding to InPO_x_ phases are detected, suggesting that the InPO_x_ coating is amorphous in nature. To further examine the phase composition, Raman spectroscopy was conducted on the pristine and InPO_x_-coated TiO_2_ NWs with varying InCl_3_ concentrations (0.05, 0.1, and 0.15 M), as shown in Figure 2b. All samples exhibit five characteristic Raman bands of the anatase phase at 145, 198, 395, 515, and 635 cm^−1^, corresponding to the Eg, Eg, B1g, A1g (or B1g), and Eg vibrational modes, respectively [12]. In addition, three weaker Raman peaks are observed at 230, 450, and 610 cm^−1^, which can be attributed to the rutile phase, corresponding to a multiphonon process, Eg mode, and A1g mode, respectively. These findings are consistent with the XRD results and confirm the coexistence of anatase and rutile phases in the TiO_2_ NWs, with the InPO_x_ layer remaining amorphous.

**Figure 3:**
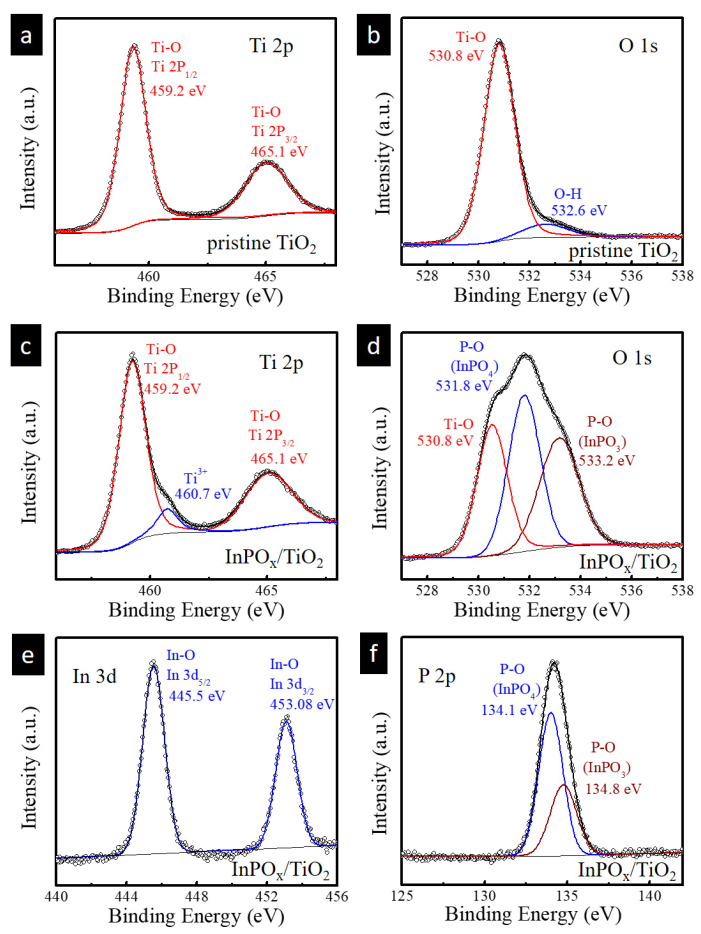
(**a**) Ti 2p and (**b**) O 1s XPS spectra of pristine TiO_2_ NWs. (**c**) Ti 2p, (**d**) O 1s, (**e**) In 3d, and (**f**) P 2p of InPO_x_/TiO_2_ NWs with 0.1 M InCl_3_ concentration.

To elucidate the oxidation states of the pristine TiO_2_ and InPO_x_/TiO_2_ NWs (prepared with 0.1 M InCl_3_), X-ray photoelectron spectroscopy (XPS) analysis was performed. Figure 3a,b show the high-resolution XPS spectra of Ti 2p and O 1s for the pristine TiO_2_ NWs. Two prominent peaks located at 459.2 eV and 465.1 eV are assigned to Ti 2p_3_/_2_ and Ti 2p_1_/_2_, respectively. The spin–orbit splitting of approximately 6.0 eV is consistent with the presence of Ti^4+^, confirming the normal oxidation state of Ti in TiO_2_, which is in agreement with previously reported data [13]. The O 1s spectrum of the pristine TiO_2_ NWs can be deconvoluted into two components: a peak at 530.8 eV attributed to the lattice oxygen in Ti–O bonds, and a peak at 532.6 eV corresponding to surface-adsorbed hydroxyl groups (–OH). Upon deposition of the InPO_x_ overlayer, an additional Ti^3+^ 2p_3_/_2_ peak appears at 460.7 eV, suggesting the generation of oxygen vacancies during the thermal phosphidation process [13]. The O 1s spectrum of the InPO_x_/TiO_2_ NWs also reveals two additional peaks at 531.8 eV and 533.2 eV, which are ascribed to P–O bonds in InPO_4_ and InPO_3_, respectively. These findings support the formation of phosphate-containing species on the TiO_2_ surface. Furthermore, the high-resolution In 3d XPS spectrum shows two distinct peaks at 445.5 eV and 453.0 eV, corresponding to In 3d_5_/_2_ and In 3d_3_/_2_ of the InPO_x_ species, respectively. The P 2p spectrum exhibits peaks at 134.1 eV and 134.8 eV, which are consistent with the 2p orbital binding energies of PO_3_^3−^ and PO_4_^3−^ species, respectively [14]. These results collectively confirm the successful formation of an InPO_x_ shell on the surface of the TiO_2_ NWs through the phosphidation process.

**Figure 4:**
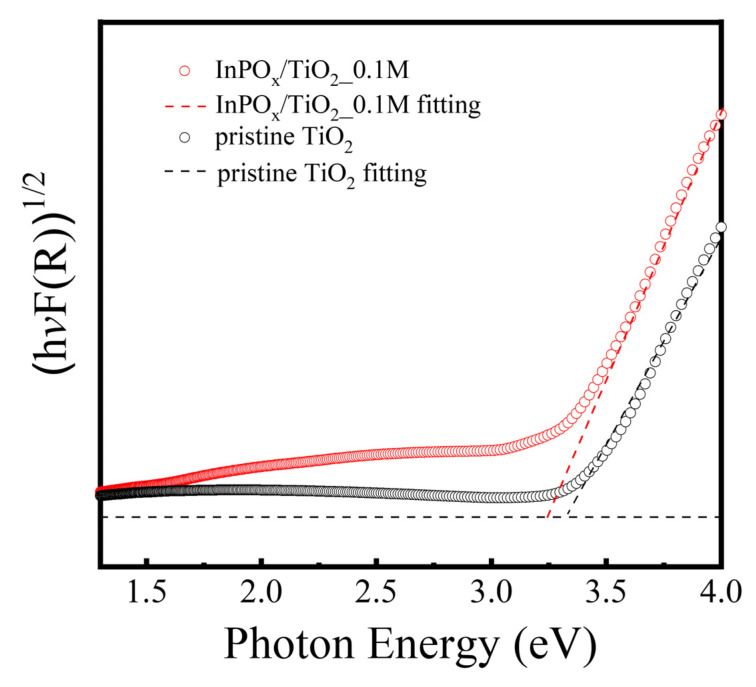
Absorption spectra of pristine TiO_2_ and InPO_x_/TiO_2_ NWs with 0.1 M InCl_3_ concentration.

In addition, the optical absorption properties of the pristine TiO_2_ and InPO_x_/TiO_2_ NWs were investigated, as shown in Figure 4. The indirect Tauc plots, derived from (αhv)^1/2^ versus photon energy (hv), were used to estimate the optical bandgap values of the two samples. Both samples exhibit a bandgap of approximately 3.2 eV, which is consistent with the reported value for anatase TiO_2_ [15]. Since InPO_x_ is a wide bandgap material, its presence as a surface shell does not significantly affect the light absorption characteristics of the TiO_2_ NWs. This confirms that the core TiO_2_ maintains its photoresponsive behavior, while the InPO_x_ layer provides surface modification without impeding optical performance.

### 2.2. PEC Performance of InPO_x_/TiO_2_ NW Photoanode

**Figure 5:**
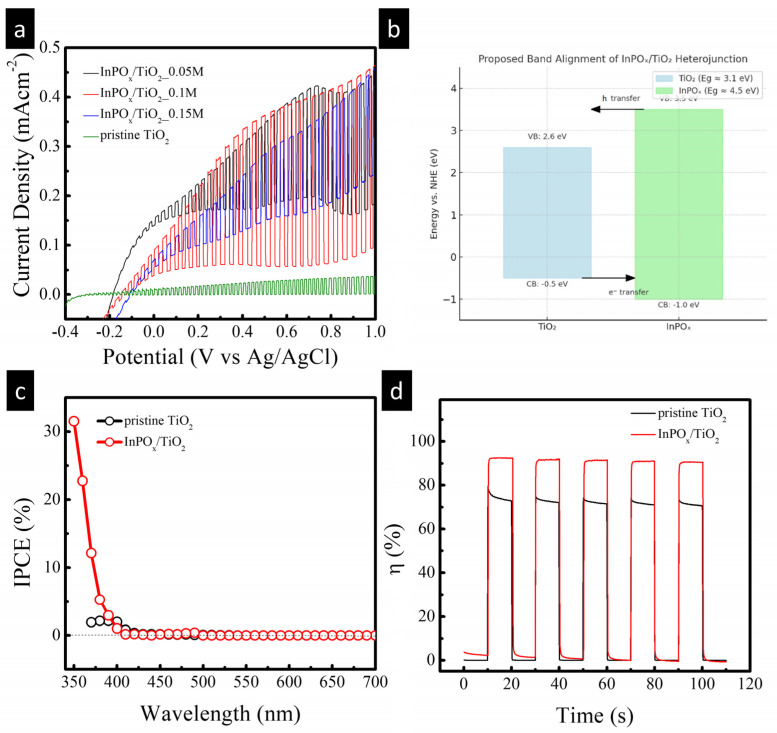
(**a**) Linear sweep voltammetry under illumination of InPO_x_/TiO_2_ NWs with different InCl_3_ concentrations. (**b**) Band edge positions of amorphous InPO_4_ and TiO_2_. (**c**) IPCE and (**d**) injection efficiency of pristine TiO_2_ and InPO_x_/TiO_2_ samples with 0.1 M InCl_3_ concentration.

To evaluate the photoelectrochemical (PEC) activity of the InPO_x_/TiO_2_ NW electrodes, a standard three-electrode configuration was employed in a 0.5 M Na_2_SO_4_ electrolyte. Photocurrent–potential (J–V) curves of pristine TiO_2_ NWs and InPO_x_/TiO_2_ NWs prepared with InCl_3_ concentrations of 0.05, 0.1, and 0.15 M were measured using linear sweep voltammetry (LSV) under both continuous and chopped illumination, as shown in Figure 5a. The pristine TiO_2_ NWs displayed a relatively low photocurrent density of approximately 0.035 mA·cm^−2^ at 0.1 V, which is primarily attributed to inefficient charge separation. Upon deposition of the InPO_x_ layer, all modified samples exhibited a significant enhancement in photocurrent response. This improvement is attributed to the formation of an InPO_x_/TiO_2_ heterojunction, which facilitates the separation of photogenerated electron–hole pairs via an internal electric field across the interface. Among the tested samples, the InPO_x_/TiO_2_ NWs synthesized with 0.1 M InCl_3_ showed the highest photocurrent density of 0.36 mA·cm^−2^ at 1.0 V—nearly an order of magnitude greater than that of the pristine TiO_2_. This enhancement corresponds to a current gain of approximately 928%, which surpasses the performance of many previously reported TiO_2_-based heterostructured photoanodes, as summarized in Table 1 [16,17,18,19,20,21]. Based on reported band edge positions of amorphous InPO_4_ (bandgap ~4.5 eV, CB ≈ −1.0 eV vs. NHE, VB ≈ +3.5 eV) and TiO_2_ (bandgap ~3.1 eV, CB ≈ −0.5 eV vs. NHE, VB ≈ +2.6 eV), we propose a type-II band alignment at the InPO_x_/TiO_2_ interface. In this configuration, photogenerated electrons in the TiO_2_ conduction band remain available for reduction reactions, while the InPO_x_ layer provides surface states that facilitate hole transfer to the electrolyte and suppress recombination. A schematic band alignment has been added to the revised manuscript (Figure 5b) to illustrate this mechanism and clarify the role of the InPO_x_ shell in enhancing charge separation. The incident photon-to-current conversion efficiency (IPCE) spectra of the pristine TiO_2_ and InPO_x_/TiO_2_ photoanodes are presented in Figure 5c. Measurements were conducted at an applied bias of 0.6 V. Both samples exhibit photocurrent onset at wavelengths around 400 nm, consistent with the bandgap of TiO_2_ [12]. However, the InPO_x_-modified sample demonstrates a marked increase in IPCE across the UV region, indicating that the InPO_x_ layer enhances ultraviolet light utilization and thus contributes meaningfully to the overall photocurrent. To further assess the charge injection capability of the InPO_x_/TiO_2_ electrode, 0.2 M Na_2_SO_3_ was added as a hole scavenger to the 0.5 M Na_2_SO_4_ electrolyte. The injection efficiency (η) was calculated using the following relation:

*η* = *j* _electrolyte_/*j* _scavenger_ (1)

where *j*_electrolyte_ is the photocurrent density measured in the scavenger-free electrolyte and *j*_scavenger_ is the photocurrent measured in the presence of the hole scavenger. As shown in Figure 5d, the injection efficiency of the InPO_x_/TiO_2_ NWs (0.1 M InCl_3_) reached approximately 91%, significantly higher than that of pristine TiO_2_. This enhancement confirms that the amorphous InPO_x_ layer functions as an effective surface cocatalyst, promoting hole transfer to the electrolyte and improving overall charge injection efficiency, consistent with its role discussed in the earlier compositional analysis [22,23].

**Figure 6:**
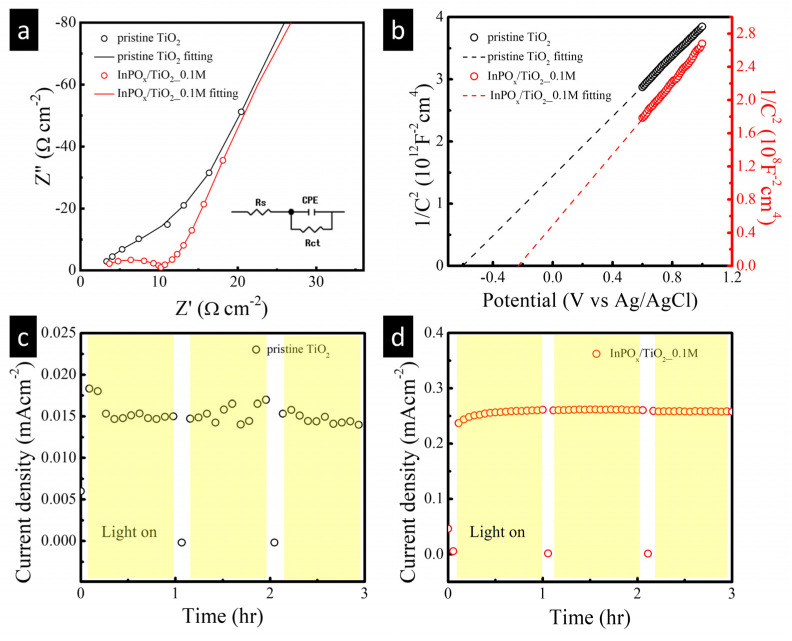
(**a**) Nyquist plots and (**b**) Mott–Schottky plot of pristine TiO_2_ and InPO_x_/TiO_2_ samples. Stability test of (**c**) pristine TiO_2_ and (**d**) InPO_x_/TiO_2_ samples.

Furthermore, electrochemical impedance spectroscopy (EIS) was performed under light irradiation at an applied potential of 0.6 V to investigate the interfacial charge transfer kinetics. As shown in Figure 6a, the Nyquist plots of the pristine TiO_2_ and InPO_x_/TiO_2_ NW photoelectrodes reveal a significantly smaller semicircular arc for the InPO_x_-modified sample. This indicates a lower charge transfer resistance and suggests more efficient interfacial charge transfer in the InPO_x_/TiO_2_ NWs. These results confirm that the incorporation of the InPO_x_ shell enhances the photoelectrochemical performance by facilitating faster charge transport at the electrode–electrolyte interface. To further understand the electronic properties, Mott–Schottky (M–S) analysis was conducted (Figure 6b). The carrier densities, estimated from the slopes of the M–S plots, were 6.9 × 10^17^ cm^−3^ for the pristine TiO_2_ NWs and 7.5 × 10^21^ cm^−3^ for the InPO_x_/TiO_2_ NWs. This nearly four-order-of-magnitude increase in carrier density highlights the significant impact of the InPO_x_ overlayer in enhancing charge carrier concentration. Such a substantial enhancement is attributed to the InPO_x_ shell on the TiO_2_ NW surface, and similar phenomena have also been observed in other InPO_x_/β-In_2_S_3_ heterostructured photoelectrodes [10]. The increased carrier density likely contributes to improved charge separation and transport, both critical factors for PEC performance. Long-term stability tests were also conducted under continuous simulated solar illumination at an applied potential of 0.6 V for 3 h. As shown in Figure 6c, the photocurrent of the pristine TiO_2_ electrode quickly stabilized after approximately 20 min. By contrast, the InPO_x_/TiO_2_ NW photoelectrode maintained a steady photocurrent throughout the entire 3 h period, indicating excellent operational stability. This sustained photocurrent not only confirms the robustness of the InPO_x_/TiO_2_ heterostructure under prolonged illumination but also implies that the InPO_x_ shell promotes efficient charge separation at low onset potentials. As a result, the photogenerated electrons more effectively drive the hydrogen evolution reaction, contributing to the enhanced photoresponse observed in the InPO_x_/TiO_2_ NW photoanode.

## 3. Experimental Section

Potassium hydroxide (KOH, 97%), indium trichloride (InCl_3_, 99%), and sodium hypophosphite (NaH_2_PO_2_, 98%) were obtained from Sigma-Aldrich (St. Louis, MO, USA). Ti foil was purchased from Shanghai Sinopharm Chemical Reagent Co., Ltd. (Shanghai, China). Hydrogen chloride (HCl, 30–50%) and sodium sulfate (Na_2_SO_4_, 98%) were purchased from Fisher Scientific (Pittsburgh, PA, USA). All chemicals were of analytical grade and used as received without further purification.

The preparation of the InPO_x_/TiO_2_ composite began with the growth of a TiO_2_ nanowire (NW) array on a Ti foil substrate using a hydrothermal method. A piece of Ti foil (0.127 mm thick, 1 cm × 2 cm) was ultrasonically cleaned in deionized water and acetone for 10 min each, then placed at an angle against the wall of a 150 mL Teflon-lined stainless-steel autoclave containing 30 mL of 1 M KOH solution. The autoclave was sealed and maintained at 220 °C in an electric oven for 48 h, resulting in the growth of potassium titanate (K_2_Ti_2_O_5_) NWs on the Ti foil. After cooling to room temperature, the sample was removed and immersed in 100 mL of 0.5 M HCl solution for 2 h to replace K^+^ ions with H^+^, thereby converting the potassium titanate NWs into hydrogen titanate (H_2_Ti_2_O_5_) NWs. The foil was then thoroughly rinsed with deionized water. In the next step, the H_2_Ti_2_O_5_ NW-covered Ti foil was annealed at 700 °C for 1 h to convert the NWs into crystalline TiO_2_ NWs.

The InPO_x_ layer was subsequently deposited on the TiO_2_ NWs by a dip-coating and thermal annealing process. Specifically, 40 μL of InCl_3_ solution at varying concentrations (0.05, 0.1, or 0.15 M) was spin-coated onto the TiO_2_ NWs. After drying, the samples were placed on a preheated hotplate at 100 °C in air for 1 min. This deposition and heating step was defined as one cycle, and ten such cycles were performed. The resulting sample was then placed in the center of a three-zone tube furnace, with 0.1 g of NaH_2_PO_2_ added to both ends. After purging the system with N_2_, the central zone of the furnace was heated to 425 °C and held for 1 h, while both ends were raised to 475 °C. The furnace was then allowed to cool naturally to room temperature, yielding the final InPO_x_/TiO_2_ composite.

The morphology of the hierarchical InPO_x_/TiO_2_ NW arrays was characterized using scanning electron microscopy (SEM, JEM-4000EX, JEOL, Tokyo, Japan). The chemical states of the constituent elements were analyzed via X-ray photoelectron spectroscopy (XPS, Perkin-Elmer PHI 1600, Shelton, CT, USA). High-resolution scanning transmission electron microscopy (HR-STEM, JEM2010F and JEM2200FS, both operating at 200 kV, JEOL) was employed to investigate the microstructure and chemical composition in detail. Photoelectrochemical (PEC) measurements were conducted in 0.5 M Na_2_SO_4_ solution using a potentiostat/galvanostat (CHI 6273D, Shanghai Chenhua Instruments Co., Ltd., Shanghai, China). A conventional three-electrode configuration was used, comprising the InPO_x_/TiO_2_ electrode as the working electrode, a square platinum sheet (4 cm^2^) as the counter electrode, and a Ag/AgCl electrode in 3 M KCl as the reference. All potentials in this study are reported versus the Ag/AgCl reference electrode. A 150 W Xe lamp equipped with an AM 1.5 filter provided simulated sunlight with an intensity of 100 mW·cm^−2^ at the sample position. A monochromator (also equipped with a 150 W Xe lamp) was used to deliver monochromatic illumination for incident photon-to-current conversion efficiency (IPCE) measurements. Light was directed onto the InPO_x_/TiO_2_ NW electrodes from the front side, passing through a quartz window and the electrolyte. Electrochemical impedance spectroscopy (EIS) was performed at a frequency of 100 kHz with an AC amplitude of 10 mV under varying applied potentials.

## 4. Conclusions

In summary, a novel InPO_x_/TiO_2_ NW photoanode was developed by integrating an amorphous indium phosphate overlayer onto hydrothermally synthesized TiO_2_ nanowires. The heterostructure design significantly enhanced photoelectrochemical performance through multiple synergistic effects, including improved charge separation, increased carrier density, and reduced charge transfer resistance. Among the fabricated samples, the InPO_x_/TiO_2_ NWs prepared using 0.1 M InCl_3_ exhibited a nearly 10-fold enhancement in photocurrent compared to pristine TiO_2_, as well as excellent operational stability over extended illumination. Mott–Schottky and EIS results confirmed the role of the InPO_x_ shell as a cocatalyst that facilitates charge transport and interfacial reaction kinetics. This work demonstrates that amorphous InPO_x_ is a promising surface modification material for constructing highly active and stable TiO_2_-based photoanodes, offering valuable insights for the rational design of advanced materials for solar energy conversion.

## Figures and Tables

**Table 1:** Comparison with studies regarding wide bandgap overlayer/TiO_2_ photoanode.

Material Structure	Nanocomposite Photocurrent Density (mA/cm^2^)	BareTiO_2_ Photocurrent Density (mA/cm^2^)	Current Density Gain Ratio	Ref.
Au@CdS/RGO/TiO_2_	0.14	0.06	133%	[16]
Al_2_O_3_/TiO_2_	~0.2	~0.02	900%	[17]
ZnO/TiO_2_	2.37	0.68	248%	[18]
SrTiO_3_/TiO_2_	~0.025	~0.012	108%	[19]
SrTiO_3_/TiO_2_	3.48	1.5	132%	[20]
RGO/TiO_2_	2.45	1.92	27%	[21]
InPOx/TiO_2_	0.36	0.035	928%	This work

## Data Availability

The data are contained within the article.
